# The Youngest Korean Case of Urachal Carcinoma

**DOI:** 10.1155/2015/707456

**Published:** 2015-06-04

**Authors:** Seung Ryeol Lee, Haeyoun Kang, Moon Hyung Kang, Young Dong Yu, Chang Il Choi, Kyung Hwa Choi, Dong Soo Park, Young Kwon Hong

**Affiliations:** ^1^Department of Urology, CHA Bundang Medical Center, CHA University, Seongnam 463-712, Republic of Korea; ^2^Department of Pathology, CHA Bundang Medical Center, CHA University, Seongnam 463-712, Republic of Korea

## Abstract

Urachal anomalies are relatively uncommon and result from incomplete obliteration of the urachus perinatally. In children, most urachal diseases including urachal cysts and sinuses are benign, and these can sometimes become secondarily infected. Malignant involvement of the urachus is rarely reported, one in 5 million people, accounting for 0.35% to 0.7% of all bladder cancers. There are only five cases of urachal cancer diagnosed at the age of twenties in English written literature. Age at the diagnosis of urachal carcinoma is important to understand pathogenetic transition from benign to malignancy. A 26-year-old man visited our clinic with gross hematuria starting a few months before. CT scan showed a 4.0 × 6.8 cm sized lobulated cystic mass over the bladder dome. Cystoscopy showed a ball-shaped extrinsic mass from the bladder dome with intact bladder mucosa. With an impression of urachal cancer, laparoscopic partial cystectomy with wide excision of urachus was performed. Final diagnosis was well differentiated mucinous urachal adenocarcinoma invading bladder muscle, staged as pT3a based on Sheldon's staging system. To our best knowledge, this case is the youngest Korean case of urachal carcinoma (the fourth youngest ever in English written literature).

## 1. Introduction

The urachus is a fibrous remnant of the allantois which is a canal draining the urinary bladder of the fetus, joining and running within the umbilical cord. The natural disease course of urachal remnants is unknown and rarely studied. If urachal remnants are not removed in childhood and they are observed, exposure to chronic urinary stasis, infection, and inflammation may predispose the patient to potential urachal carcinoma. Urachal carcinoma is a rare malignancy accounting for less than 1% of bladder cancers. It is especially rare in young adults while most cases are occurring from middle to old age. This can be one of the clues for pathogenetic transition from benign urachal anomaly to urachal carcinoma.

## 2. Case Report

**Figure 1:**
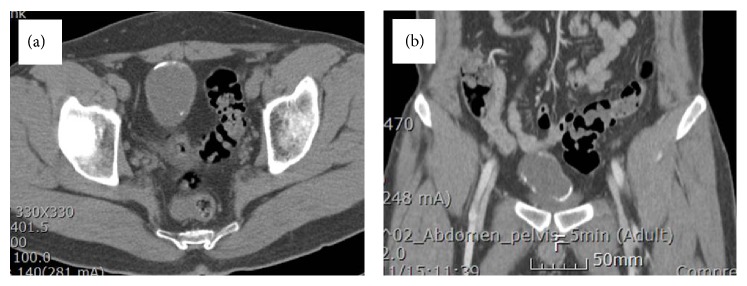
CT scan shows a lobulated cystic mass over the bladder dome which has calcified thick wall and is located at midline to the right side.

**Figure 2:**
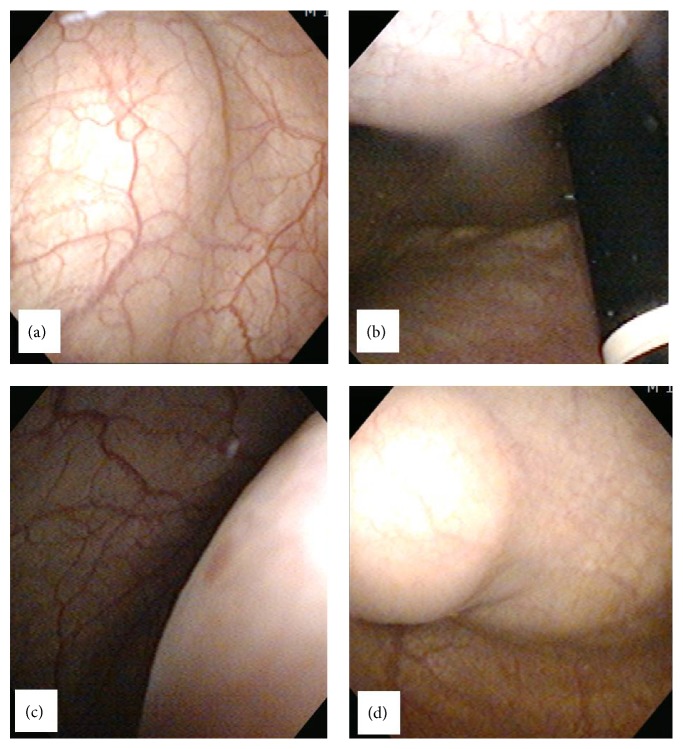
Cystoscopy shows a ball-shaped extrinsic mass from the bladder dome with intact bladder mucosa.

**Figure 3:**
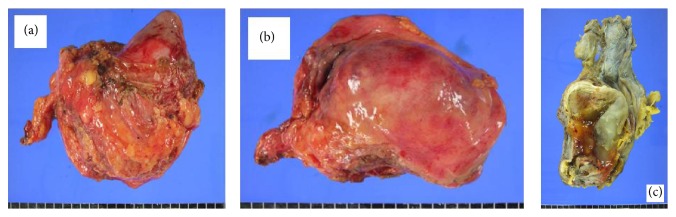
Surgical specimen shows a unilocular cyst without definite solid part, filled with gray tan to brownish thick mucus material.

A 26-year-old man visited our clinic with gross hematuria starting a few months before. Urinalysis showed many RBC, and CT scan showed a 4.0 × 6.8 cm sized lobulated cystic mass over the bladder dome which was located midline to the right side. The mass has calcified thick wall with suspicious contrast enhancement (Figure 1). Cystoscopy showed a ball-shaped extrinsic mass from the bladder dome with intact bladder mucosa (Figure 2). With an impression of urachal cancer, laparoscopic surgery was performed. Camera port was placed 5 cm above the umbilicus, and two instrument ports were placed 7 cm lateral and 2 cm caudal to the camera port. Anterior peritoneum was dissected deep and widely from the level of umbilicus down to the UB including median umbilical ligament and part of both medial umbilical ligaments. Cystoscope was introduced from the urethra into the bladder to illuminate the margin of the mass and to facilitate keeping enough distance from the mass during laparoscopic partial cystectomy. Bladder was repaired continuously with 4-0 vicryl suture. After placing a closed suction drain the mass was wrapped into an Endo Catch bag and squeezed out through the extended umbilical opening. A Foley catheter was inserted into the bladder and the port sites were closed. Cross section of the surgical specimen showed a unilocular cyst without definite solid part, filled with gray tan to brownish thick mucus material. It also showed whitish to yellowish thick wall with multifocal calcifications (Figure 3). Immunohistochemical staining was strong positive on CEA, positive on CD15 (LeuMI), and weak positive on CK7 and CK20. Final diagnosis was well differentiated mucinous urachal adenocarcinoma invading bladder muscle, pT3a based on Sheldon's staging system, pT2b based on Mayo system, and pT2 based on Ontario system. Surgical margin was negative.

## 3. Discussion 

Malignant involvement of the urachus is rarely reported, one in 5 million people, accounting for 0.35% to 0.7% of all bladder cancers [1]. It has been reported mostly in adults over 30 years old. To our best knowledge, only seven cases of urachal carcinoma diagnosed before the age of 30 have been reported in the English written literature including two cases occurring in two 15-year-old girls [2–7]. Even in a large series study of Mayo clinic including 49 cases, the youngest patient was 43 years old [8]. The youngest adult (22 years old) case of urachal cancer was reported by Siefker-Radtke et al. from MD Anderson Cancer Center [4]. According to the MSKCC study of 24 cases, two young men, who were 26 and 29 years old, respectively, were found to have urachal cancer [5]. The 26-year-old man received extended partial cystectomy, umbilectomy, and pelvic LN dissection and survived at 0.9-month follow-up without long-term survival data. The 29-year-old man received extended partial cystectomy and umbilectomy and survived at 17-month follow-up without local recurrence or metastasis. Our case is an urachal adenocarcinoma occurring in a 26-year-old man, the youngest case in Korea and the fourth-youngest case ever reported in English written literature.

The criteria for the diagnosis of urachal carcinoma are somewhat controversial, and different staging systems for urachal carcinoma have been proposed: the Sheldon system [1], the Mayo systems [8], and the Ontario systems [6]. Sheldon proposed a staging system as follows: pT1, no invasion beyond the urachal mucosa; pT2, invasion confined to the urachus; pT3, local extension to the (a) bladder, (b) abdominal wall, and (c) viscera other than the bladder; and pT4, metastasis to (a) regional lymph nodes and (b) distant sites. Mayo clinic implemented the TNM staging system and proved that anatomic location can be extrapolated to prognosis after surgical resection. The TNM staging system that they used is as follows: Tis is a tumor localized to the urachal mucosa with no invasion to the basal membrane (carcinoma in situ). T1 is a tumor with invasion through the basal membrane. T2 is divided into pT2a (deep) and pT2b (superficial) according to muscle invasiveness. T3 is a tumor invading perivesical fat or abdominal wall muscle (in cases of extravesical urachal tumors). Involvement of regional lymph nodes followed the traditional TNM staging system. Peritoneal implants were considered metastasis or stage IV. Ontario group classified the primary tumor as T1 when a tumor was confined to the submucosa, T2 when a tumor was confined to the muscular wall of the bladder, T3 when a tumor extended into the periurachal or vesical soft tissue, and T4 when a tumor invades adjacent organs, including the abdominal wall. Therefore this case can be staged differently as pT3a based on Sheldon's staging system, pT2b based on Mayo system, and pT2 based on Ontario system. At any rate, this case is considered curable.

Treatment for urachal cancer is focused on surgical excision. Wide excision with negative margins must be achieved to minimize the risk of local recurrence, for which no effective salvage therapy exists. The appropriate type of operation however remains controversial (radical versus partial cystectomy, open versus laparoscopic). As the survival benefit of radical over partial cystectomy is difficult to assess due to the absence of controlled studies and the limited experience reported, the surgical approach has leaned more towards bladder sparing. Therefore, laparoscopic partial cystectomy can be one of the options along with open surgery for the treatment of urachal cancer. Laparoscopy has the advantage of providing excellent surgical field and adequate resection margins with the aid of magnification and cystoscopic lighting or tattooing [9].
